# Clear Cell Meningioma in the Central Nervous System: Analysis of Surveillance, Epidemiology, and End Results Database

**DOI:** 10.3389/fonc.2020.592800

**Published:** 2021-01-22

**Authors:** Yubo Wang, Xiaowei Qin, Mingyang Liu, Xinrui Liu, Ying Yu, Gang Zhao, Ying Xu

**Affiliations:** ^1^ Department of Neurosurgery, First Affiliated Hospital of Jilin University, Changchun, China; ^2^ College of Instrument Science and Electrical Engineering, Jilin University, Changchun, China; ^3^ Computational Systems Biology Lab, Department of Biochemistry and Molecular Biology, University of Georgia, Athens, GA, United States

**Keywords:** CNS disease, SEER Program, survival, epidemiology, clear cell meningioma

## Abstract

**Background:**

Clear cell meningioma (CCM) is a rare subtype of meningioma, accounting for approximately 0.2% of all meningiomas. The present study aimed to analyze the epidemiology and outcome of CCMs using the Surveillance, Epidemiology, and End Results (SEER) database.

**Methods:**

Patients diagnosed with central nervous system CCM between 2004 and 2016 were identified from the SEER database. Descriptive analyses were performed to evaluate the distribution of patients and tumor-related characteristics. The survival analysis was performed using Kaplan-Meier curves. The Cox proportional hazards model was used for the univariate and multivariate analyses.

**Results:**

The age-adjusted incidence rate was 0.032 per 1,000,000 person-years. The median age was 52 years. Most of the CCMs were intracranial CCMs that were larger than 3 cm. The overall cumulative survival rates at 1, 3, and 5 years were 97.6, 93.2, and 86.9%, respectively. The log‐rank test and Cox proportional hazards regression analysis revealed that age at diagnosis and primary site of the tumor were independent prognostic factors.

**Conclusion:**

CCM is an extremely rare entity with a favorable survival rate. CCMs usually affect patients during the fourth to fifth decades of life. Patients diagnosed at 21–60 years old and patients with spinal CCMs have a better prognosis.

## Introduction

Clear cell meningioma (CCM) is an exceedingly rare variant of meningioma characterized by sheets of round or polygonal cells with a clear and glycogen-rich cytoplasm, and prominent perivascular and interstitial collagen. CCMs predominantly affect children and young adults (1). Furthermore, CCM has been recognized by the World Health Organization as a Grade II neoplasm, and aggressive clinical course and occasional cerebrospinal fluid metastasis have been widely reported (1, 2). The existing literature on CCM is limited to case reports and small case series. Due to the rarity of CCMs, the epidemiological and clinical characteristics of CCMs have not been fully understood. The Surveillance, Epidemiology, and End Results (SEER) program of the National Cancer Institute collects data on tumor diagnoses (including the primary site and tumor morphology), treatment, and survival for approximately 34.6% of the United States (US) population (3). The present study aimed to analyze the epidemiology and outcome of CCMs using the SEER database.

## Methods

### Data Extraction

The SEER database is available to the public for research purposes, and no ethics committee approval or informed consent is required. The age-adjusted incidence rates (directly standardized to the 2000 US standard million population) between 2004 and 2016 were calculated using the SEER 18 database (November 2019 submission) (4). The detailed patient demographic data and clinical profiles were obtained from the SEER 18 Regs Custom Data (November 2018 submission) (5). The diagnosis of “central nervous system clear cell meningioma” was defined using the International Classification of Diseases for Oncology 3 (ICD-O-3) code 9538/0 and 9538/1, and the site was set as “brain and other nervous system.” Since the SEER program has identified benign and borderline tumors of the central nervous system since 2004, the time span of the diagnosis was set as 2004–2016. Only the patients diagnosed with positive histology according to the code “Diagnostic Confirmation” were included in the population analysis. Additionally, only the patients with active follow-up according to the code “Type of follow-up expected” were included for the survival analysis. All data were obtained using the SEER*Stat 8.3.6 software. Statistical analyses were performed using the SPSS Statistics 26.0 software (IBM Corporation, Armonk, NY, USA).

### Population Analysis

The demographic and clinical variables included gender (male or female), age (0–20, 21–40, 41–60, or >60 years old), race (white, black, or others), primary tumor site (cerebral meninges, spinal meninges, or not clarified), behavior code ICD-O-3 (benign or borderline malignancy), tumor size (≤3 cm, >3 cm, or unknown), radiation (yes or no/unknown), and the extent of the surgical resection. The investigators categorized the surgical procedure codes into three groups, as previously described (6, 7): no surgery (code 00), partial resection (20, 21, 22, 30, and 40), and gross total resection (code 55). Descriptive analyses were conducted to evaluate the distribution of patients and tumor-related characteristics. The age distribution of the patients at diagnosis was described using a histogram.

### Survival Analysis

The Kaplan-Meier curve analysis was performed to estimate the overall survival (OS), and the intergroup differences were assessed using log‐rank tests. All variables with a significant result in the univariate Cox proportional hazard regression analysis were included in the following multivariate analysis. The hazard ratios (HR) and 95% confidence intervals (CIs) were estimated to identify the independent prognostic factors associated with OS in patients with CCM. A *P*-value of ≤0.05 was considered statistically significant.

## Results

### Population Analysis

**Figure 1:**
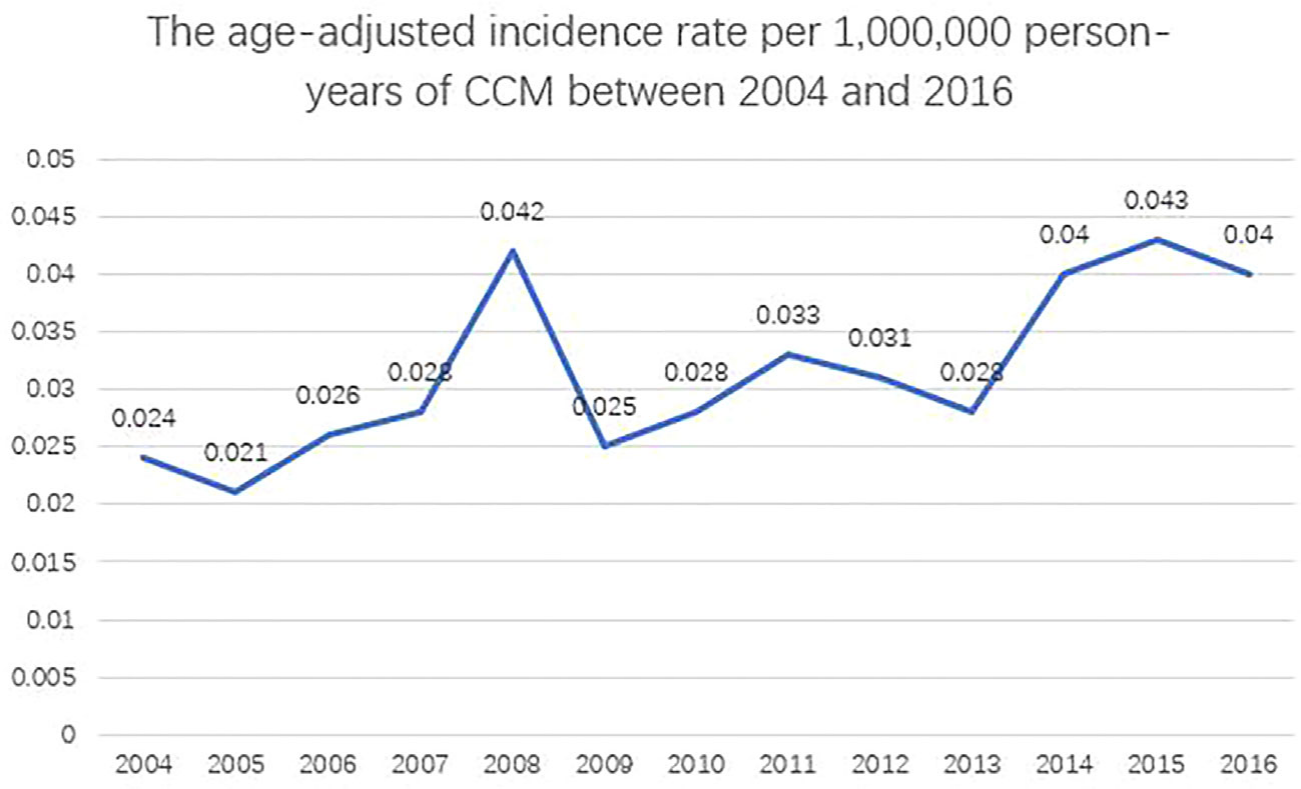
The age-adjusted incidence rates of the central nervous system clear cell meningioma between 2004 and 2016.

**Figure 2:**
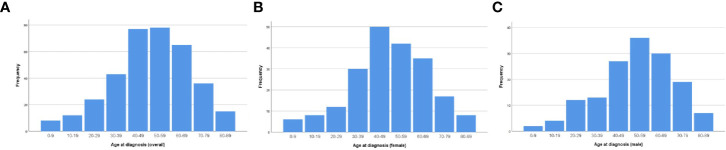
Age distribution of the patients at diagnosis: **(A)** for the whole cohort; **(B)** for female patients; **(C)** for male patients.

The age-adjusted incidence rate was 0.031 per 1,000,000 person-years between 2004 and 2016. The annual age-adjusted incidence rate was 0.024 per 1,000,000 person-years in 2004, which increased to 0.040 per 1,000,000 person-years in 2016 (**Figure 1**). The age-adjusted incidence rate for male is 0.027 per 1,000,000 person-years. Compared to male counterpart, the rate for female is 0.036 per 1,000,000 person-years, and the rate ratio and 95% CI is 1.3195 (1.0613–1.6437). A total of 363 cases of CCM in the central nervous system were identified between 2004 and 2016. There were 358 cases diagnosed with positive histology in the population analysis. The demographic and clinical characteristics of these patients are summarized in **Table 1**. There were 208 female patients (58.1%) and 150 male patients (41.9%), yielding a female-to-male ratio of 1.4:1.0. The average age was 50.77 ± 17.648 years (median, 52 years; range, 3–88 years). Among all these patients, 41.9% of these patients (n = 150) were within 41–60 years old, and 74.9% of these patients were white (*n* = 268). CCMs most commonly affect patients during the fourth to sixth decades of life (**Figure 2A**). When the female and male patients were analyzed separately, we found that CCMs usually occur during the fourth to fifth decades of life in female patients (**Figure 2B**) and during the fifth to sixth decades of life in male patients (**Figure 2C**). Most of the tumors were borderline malignancy (n = 350, 97.8%). The primary tumor site was available for 326 patients. The tumor arose from the cerebral meninges in 285 patients (87.4%), and from the spinal meninges in 41 patients (12.6%). The tumor size was available in 272 patients, and the tumor size was larger than 3 cm in 207 patients (76.1%). Surgical resection was performed for 342 (95.5%) patients. Among these patients, partial resection was achieved in 271 patients (75.7%), and gross total resection was achieved in 71 patients (19.8%). Radiation therapy was performed for 101 (28.2%) patients. At the time of data collection, 304 (84.9%) patients were alive and 54 (15.1%) were deceased.

**Table 1:** Demographic and clinical characteristics of patients with CCM.

Variables	Number	%
**Sex (n = 358)**		
Female	208	58.1
Male	150	41.9
**Age at diagnosis (years; n = 358)**		
Mean ± SD	50.77 ± 17.648	
Median	52	
Range	3–88	
0–20	21	5.9
21–40	75	20.9
41–60	150	41.9
>60	112	31.3
**Primary Site (n = 326)**		
Cerebral meninges	285	87.4
Spinal meninges	41	12.6
**Race (n = 358)**		
White	268	74.9
Black	52	14.5
Others	38	10.6
**Behavior code ICD-O-3 (n = 358)**		
Benign	8	2.2
Borderline malignancy	350	97.8
**Tumor Size (n = 272)**		
≤3cm	65	23.9
>3cm	207	76.1
**Extent of resection (n = 358)**		
No resection	16	4.5
partial resection (PR)	271	75.7
gross total resection (GTR)	71	19.8
**Radiation (n = 358)**		
Yes	101	28.2
None/Unknown	257	71.8
**Vital status (n = 358)**		
Alive	304	84.9
Dead	54	15.1

### Survival Analysis

**Figure 3:**
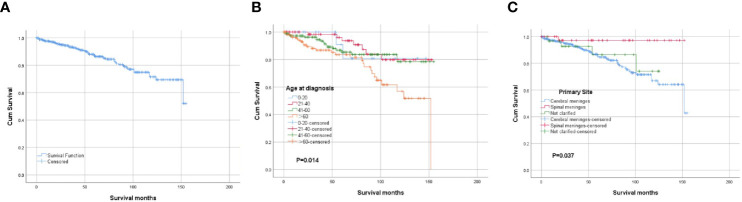
Kaplan-Meier survival analysis: **(A)** The overall survival for the whole cohort. **(B)** The survival analysis of patients classified based on the age at diagnosis. **(C)** The survival analysis of patients classified based on the primary tumor site.

The overall cumulative survival rates at 1, 3, and 5 years were 97.6, 93.2, and 86.9%, respectively. The OS of the whole cohort was presented as a Kaplan-Meier curve in **Figure 3A**. The log‐rank tests indicated that age at diagnosis (**Figure 3B**) and primary tumor site (**Figure 3C**) were the potential risk factors for OS. The univariate analyses revealed that patients diagnosed at 21–60 years old and patients with spinal tumors had a more favorable prognosis. The codes of age at diagnosis and primary site were included in the multivariate analysis. The multivariate Cox proportional hazards regression analysis revealed that age at diagnosis (21–60 years old) was an independent factor for predicting a favorable prognosis. And the patients with spinal CCMs had better prognosis than those with intracranial CCMs. The statistical results are summarized in **Table 2**.

**Table 2:** The results of the log-rank test, and univariate and multivariate Cox regression analysis.

Variable	Log-Rank Test	Univariate Analysis		Multivariate Analysis	
	P value	HR (95% CI)	P value	HR (95% CI)	P value
**Sex**	0.658				
Female		Reference			
Male		1.132 (0.653–1.963)	0.658		
**Age at diagnosis** (years)	0.014				
0–20		0.385 (0.091–1.623)	0.193	0.688 (0.159–2.979)	0.617
21–40		0.335 (0.145–0.773)	0.010	0.370 (0.160–0.855)	0.02
41–60		0.485 (0.263–0.895)	0.021	0.505 (0.273–0.933)	0.029
>60		Reference		Reference	
**Primary Site**	0.037				
Cerebral meninges		Reference		Reference	
Spinal meninges		0.117 (0.016–0.852)	0.034	0.132 (0.018–0.988)	0.049
Not clarified		0.790 (0.284–2.196)	0.652	0.707 (0.253–1.973)	0.508
**Race**	0.866				
White		Reference			
Black		0.802 (0.357–1.802)	0.593		
Others/Unknown		0.982 (0.387–2.490)	0.969		
**Behavior code**	0.212				
Benign		Reference			
Borderline malignancy		22.325 (0.013–37245.571)	0.412		
**Tumor Size**	0.104				
≤3cm		Reference			
>3cm		2.449 (0.957–6.267)	0.062		
Unknown		1.625 (0.578–4.572)	0.357		
**Extent of resection**	0.229				
No resection		Reference			
PR		2.308 (0.314–16.979)	0.412		
GTR		3.413 (0.460–25.345)	0.230		
**Radiation**	0.831				
None/Unknown		Reference			
Yes		0.934 (0.497–1.755)	0.832		

## Discussion

CCM was first reported by Harkin et al. (8), and the electron microscopic examination of spinal meningioma demonstrated broad zones with large amianthoid collagen fibers in the tumor. CCM was previously classified as Grade I, according to the World Health Organization (WHO) Classification of Central Nervous System Tumors (2). However, due to its high recurrence rate and aggressive clinical course, CCM was later prompted to Grade II (9). According to existing literatures (10, 11), CCM represents as one of the rarest subtypes that occupies approximately 0.2–0.8% of all meningiomas, and the present evidence is limited to single case reports or small case series. Although some gene mutations have been reported to be associated with CCM, such as the neurofibromatosis gene (*NF-2*) (11, 12) and *SMARCE1* (13–15), the definitive etiology of CCM remains unclear.

Zhang et al. reviewed all the reported cases of intracranial CCMs (16) and spinal CCMs (17), and they found a significant female predilection in spinal CCMs. The female-to-male ratio was 1.4:1.0 in the present study, which is consistent with previous reports (11, 18). Louis *et al.* proposed that CCMs are more likely to affect young patients, including children and young adults (1). According to Zhang’s review, 42.9% of patients with spinal CCMs were younger than 18 years old (17), and the mean age at surgery was 24 and 32 years old for spinal and intracranial CCMs, respectively (16, 17). However, the predilection for the young population was not remarkable in some reports (18). In the present cohort, the mean age was 50.77 ± 17.648 years old (median age: 52 years old), most of the patients were diagnosed at 41–60 years old and the proportion of patients younger than 20 years old was only 5.9%. This result is consistent with the findings reported by Cahill *et al.*, in which the mean age at diagnosis of patients with nonmalignant intracranial meningioma was 62 ± 16 years old (19).

CCMs predominantly occur at the cerebellopontine angle and spine, especially in the cauda equina region (1). In the present SEER database, the primary site of meningioma is classified as cerebral meninges or spinal meninges. Thus, it is difficult to analyze the definitive location of CCMs. Noteworthily, spinal CCMs constituted 12.6% of all CCMs in the present study, which is similar to a previous report on CCMs (11), and much higher than the proportion of spinal counterparts in other meningioma subtypes (20).

In the present study, most of the tumors were larger than 3 cm. Furthermore, 95.5% of these tumors were surgically resected, and 28.2% of the patients chose radiation therapy. The prognosis of spinal CCMs was better than that of cerebral CCMs. According to previous reviews (16, 17), the 5-year progression-free survival was 47% for patients with spinal CCM and 37% for patients with cerebral CCM. In the present cohort, the OS rate of patients with CCM at 1, 3, and 5 years after diagnosis was better than that in a previous report (11), and similar to that for patients with nonmalignant meningiomas (19). The extent of surgical resection and radiology therapy were not significantly correlated to the OS. Tao et al. (11) retrospectively reviewed 56 cases of CCMs, and found that the extent of resection was associated with the progression-free survival, but not with the OS (11). Another study that involved 36 cases of CCM also reported similar results (21). Since the prognosis of CCMs is generally favorable, and the OS of CCMs is much better than that of gliomas and other central nervous system malignant tumors, the progression-free survival should be a more valuable indicator for evaluating the prognosis. Unfortunately, the progression-free survival was not documented in the SEER database. According to previous reports, total resection was still the first choice of treatment (21), and the role of adjuvant radiotherapy in CCM remains controversial (10, 21). On the other hand, chemotherapy does not appear to have a significant role in the management of CCM (21).

There were several limitations in the present study. First, some valuable parameters were not available in the SEER database, such as the detailed information on the recurrence and quality of life. This information may be more important for patients with borderline or benign tumors. Second, the present study was retrospective in nature. Hence, some inherent biases may exist. However, to the best of our knowledge, this is the largest series of CCM, to date.

## Conclusion

CCM is an exceedingly rare entity with a relatively favorable prognosis. CCMs usually affect patients during the fourth to sixth decades of life. Patients diagnosed at 21–60 years old and patients with spinal CCMs have a better prognosis.

## Data Availability Statement

Publicly available datasets were analyzed in this study. This data can be found here: https://seer.cancer.gov/data/.

## Ethics Statement

Ethical review and approval was not required for the study on human participants in accordance with the local legislation and institutional requirements. Written informed consent for participation was not required for this study in accordance with the national legislation and the institutional requirements.

## Author Contributions

GZ and YX designed the research. YW, XQ, ML, XL, and YY performed the work and wrote the manuscript. All authors contributed to the article and approved the submitted version.

## Conflict of Interest

The authors declare that the research was conducted in the absence of any commercial or financial relationships that could be construed as a potential conflict of interest.
